# A Comprehensive Review on Neuroimmunology: Insights from Multiple Sclerosis to Future Therapeutic Developments

**DOI:** 10.3390/biomedicines11092489

**Published:** 2023-09-08

**Authors:** Lucian Eva, Horia Pleș, Razvan-Adrian Covache-Busuioc, Luca Andrei Glavan, Bogdan-Gabriel Bratu, Andrei Bordeianu, David-Ioan Dumitrascu, Antonio Daniel Corlatescu, Alexandru Vlad Ciurea

**Affiliations:** 1 Clinical Emergency Hospital “Prof. Dr. Nicolae Oblu”, 700309 Iasi, Romania; elucian73@yahoo.com; 2 Department of Neurosurgery, Centre for Cognitive Research in Neuropsychiatric Pathology (NeuroPsy-Cog), “Victor Babeș” University of Medicine and Pharmacy, 300041 Timisoara, Romania; 3 Department of Neurosurgery, “Carol Davila” University of Medicine and Pharmacy, 020021 București, Romania; luca-andrei.glavan0720@stud.umfcd.ro (L.A.G.); bogdan.bratu@stud.umfcd.ro (B.-G.B.); andrei.bordeianu@stud.umfcd.ro (A.B.); david-ioan.dumitrascu0720@stud.umfcd.ro (D.-I.D.); antonio.corlatescu0920@stud.umfcd.ro (A.D.C.); prof.avciurea@gmail.com (A.V.C.)

**Keywords:** multiple sclerosis, neuroimmunology, therapeutic development

## Abstract

This review delves into neuroimmunology, focusing on its relevance to multiple sclerosis (MS) and potential treatment advancements. Neuroimmunology explores the intricate relationship between the immune system and the central nervous system (CNS). Understanding these mechanisms is vital for grasping the pathophysiology of diseases like MS and for devising innovative treatments. This review introduces foundational neuroimmunology concepts, emphasizing the role of immune cells, cytokines, and blood–brain barrier in CNS stability. It highlights how their dysregulation can contribute to MS and discusses genetic and environmental factors influencing MS susceptibility. Cutting-edge research methods, from omics techniques to advanced imaging, have revolutionized our understanding of MS, offering valuable diagnostic and prognostic tools. This review also touches on the intriguing gut–brain axis, examining how gut microbiota impacts neuroimmunological processes and its potential therapeutic implications. Current MS treatments, from immunomodulatory drugs to disease-modifying therapies, are discussed alongside promising experimental approaches. The potential of personalized medicine, cell-based treatments, and gene therapy in MS management is also explored. In conclusion, this review underscores neuroimmunology’s significance in MS research, suggesting that a deeper understanding could pave the way for more tailored and effective treatments for MS and similar conditions. Continued research and collaboration in neuroimmunology are essential for enhancing patient outcomes.

## 1. Introduction

### 1.1. Background

Neuroimmunology is an interdisciplinary field that brings together knowledge from biology, immunology, chemistry, neurology, pathology, psychiatry, and virology to examine the intricate interrelations between the central nervous system (CNS) and immune system (IS), its interactions during various developmental stages, as well as maintaining homeostasis or responding to injuries. Neuroimmunologists primarily seek to develop strategies to treat or even prevent neuroimmunological diseases by understanding these complex interactions in depth [1].

Tradition holds that the immune system and brain operate independently, separated by the blood–brain barrier (BBB). Yet, over recent decades, these long-held beliefs have been vigorously disproved, and evidence now exists to demonstrate otherwise. Not only does the nervous system receive communication from immune cells directly, but brain signals actively regulate immune functions—resulting in inflammation occurring elsewhere outside the central nervous system [2].

An intriguing fact about neuroimmunology is that it did not receive its introduction on PubMed until 1982—coinciding with both its inaugural congress in Stresa, Italy, as well as the launch of the *Journal of Neuroimmunology* that same year. Neuroimmunology research has traditionally centered around multiple sclerosis (MS). It is essential to acknowledge, however, that immune responses can also be seen in many other conditions like Guillain–Barré syndrome (GBS), white matter diseases, psychiatric disorders, infections, trauma, and neurodegenerative diseases that are traditionally considered more “cell autonomous”.

### 1.2. Purpose and Scope of the Review

Over time, neuroimmunology has grown into an interdisciplinary field focused on understanding the complex relationship between nervous and immune systems. Thanks to extensive research in this field, significant progress has been made in uncovering mechanisms associated with various neurological conditions—multiple sclerosis (MS) being one such disorder that sheds light on this complicated interrelationship [3].

This review seeks to assess the current level of understanding in neuroimmunology, specifically regarding multiple sclerosis research. By compiling recent discoveries, clinical data, and advances in this area of science, this comprehensive overview aims to provide an in-depth knowledge of both MS’s pathophysiological mechanisms and their significance relative to other neurological conditions.

### 1.3. Methodology of Review Selection

For this comprehensive review on neuroimmunology, with particular reference to insights gained from multiple sclerosis for future therapeutic advancement, a methodical and rigorous approach was taken in selecting and assessing literature sources. A specific methodology for selecting and assessing literature sources has been devised:

(a) Search Strategy: An extensive literature search was performed using multiple electronic databases such as PubMed, Scopus, Web of Science, and Google Scholar. Our search strategy comprised pertinent keywords and phrases related to neuroimmunology, multiple sclerosis, neuroinflammation, immune system dysfunction, central nervous system therapies, as well as therapeutic interventions with no restrictions placed on publication dates, allowing a broad representation.
(b) Inclusion and Exclusion Criteria:

To ensure the quality and relevance of this review, specific inclusion and exclusion criteria were used during the selection process. Articles meeting certain criteria were selected for inclusion: studies or research articles focused on neuroimmunology; publications providing insight into pathophysiology, etiology, clinical aspects, and immunology implications related to multiple sclerosis as they apply to neuroimmunology; clinical trials/experimental/observational research investigating therapeutic developments/interventions regarding MS/related neuroinflammatory conditions; and publications available either in English or with accessible English translations to ensure comprehension.

Subsequently, certain articles were disqualified: non-peer-reviewed materials such as conference abstracts or unpublished manuscripts were removed; studies with limited relevance to neuroimmunology of multiple sclerosis or not meeting its primary objectives were disqualified, as were articles focusing solely on non-neurological immune-related disorders or general immunology without direct ties to neuroimmunology, which were specifically excluded to ensure reliability in our findings. This process ensured our results would stand up against scrutiny.

## 2. The Basics of Neuroimmunology

### 2.1. Definition and Overview

Neuroimmunology is an interdisciplinary field devoted to understanding the complex relationships and interactions between the nervous system (including the brain and spinal cord) and immune system (including antibodies and their targets) in maintaining equilibrium in our bodies and responding to infections, injuries, or diseases that affect these vital systems. Neuroimmunology offers an examination of this topic that spans numerous fields [4].

Multiple sclerosis (MS) is one of the most prevalent disabling neurological afflictions among young adults, typically appearing between 20 and 40 years of age.

MS is an autoimmune condition in which immune system cells, normally responsible for protecting against viruses, bacteria, and abnormal cells in the body, attack myelin in the central nervous system (brain, optic nerves, and spinal cord). Myelin acts as a protective substance by creating sheaths (myelin sheaths) around nerve fibers (axons).

MS is a chronic condition that varies considerably among its victims, from mild cases with limited disability to progressive decline leading to greater disability over time. Most commonly seen are intermittent symptoms surfacing followed by periods of relative quiescence or dormancy and then either partial or full recovery—MS is rarely fatal. Individuals diagnosed tend to have life expectancies comparable to the general population [5].

### 2.2. Key Players in Neuroimmunology: Cells and Molecules

As part of their shared response to environmental challenges, the nervous and immune systems have formed an interdependent communication mechanism between themselves in response to environmental challenges. Neurons exhibit various receptors found on immune cells, such as Toll-like receptors (TLRs) and inflammatory cytokine receptors found on immune cells; this allows immune cells to influence and regulate neuronal activity—for instance, using IL-1β to sensitize sensory neurons during inflammation while managing pain levels [6].

Immune cells are capable of sensing signals from neurons by expressing receptors for neurotransmitters and neuropeptides produced by neurons; for example, innate lymphoid cells express such receptors for neuropeptides as Calcitonin Gene-Related Peptide (CGRP) and Neuromedin U (NMU). This mutual sensing between immune cells and neurons has proved highly advantageous, decreasing costs related to dealing with certain insults and helping coordinate complex host responses more efficiently.

Furthermore, the microbiome—or collection of microorganisms that live inside your body—plays a key role in both neuronal activation and immune development. Immune cells and neurons both interact directly or indirectly with microbes present in the environment, making their composition key for shaping neuronal programming and maturation. In turn, this affects various aspects of intestinal physiology, including visceral pain management, gut motility regulation, and related functions. This interaction between the nervous system, immune system, and microbiome plays an intricate, evolutionary, beneficial process that plays its part in controlling host responses overall [7,8,9].

### 2.3. Neuroimmune Communication Pathways

Recent research has demonstrated that the peripheral immune system and nervous system can communicate effectively by using similar molecular signaling cues.

Veiga-Fernandes and Pachnis’ work presents the idea of an “enteric neuroimmune cell unit,” an internal sensory organ responsible for protecting and maintaining intestinal integrity and function. This enteric neuroimmune system plays an essential role in providing both innate defenses and memory responses against certain pathogens. During gestation, extracellular signals coordinate the development of enteric neuroprogenitor and hematopoietic cells, resulting in this complex network. Postnatal development of this system takes place through colonization with commensal microbes that colonize the gut, leading to mutual signals between neuronal–glial cells and tissue-resident immune cells that stimulate the maturation of an enteric neuroimmune system [10].

It is believed that gut microbiomes have an influence over peripheral immune cells and central nervous system (CNS)-residing cells, as well as having an influence over brain development and disease progression. They stress the role of commensal microbes in producing short-chain fatty acids and aryl hydrocarbon ligands that may alter glial cell development and function within the CNS. Neuroendocrine mediators produced through the hypothalamic–pituitary–adrenal axis can influence intestinal permeability, immune-cell activation, and the composition of gut microbiomes. Dysbiosis of microbiota has been observed in neurological and psychiatric conditions, suggesting it could contribute to their causes; however, more research needs to be conducted in order to uncover its exact nature and causal role.

Prinz and Priller examine how peripheral immune cells may enter the CNS under pathological circumstances. A healthy CNS does not normally contain blood-borne immune cells, as immune surveillance is provided by tissue-resident microglia, meningeal macrophages, and perivascular macrophages, which produce their own immunological mediators. In conditions like autoimmune diseases, infections, or injuries, where blood–brain barrier permeability changes, activated adaptive immune cells are allowed into CNS via fenestrated capillaries, contributing towards disease progression [11].

Engelhardt and colleagues investigate the CNS’s immunological advantages towards peripheral immune cells under normal circumstances, distinguishing between lymphatic drainage of cerebrospinal fluid that bathes meninges and cerebral ventricles from its lack of drainage in interstitial fluid bathing CNS parenchymal tissues, such as interstitial drainage. They provide a detailed anatomical account of both human and rodent brain barriers limiting access to CNS parenchyma, which prevent lymphatic drainage of cerebrospinal fluid from meninges/ventricles, while discussing both trafficking of cells/solutes through these barrier sites via their perivascular areas allowing lymphatic drainage or non-drainage channels [12].

Further research explores neurological impairments caused by acute infections caused by neurotropic pathogens. Acute infections trigger the release of pro-inflammatory cytokines by astrocytes, microglia, and leukocytes into the CNS from cells like astrocytes and microglia, which release these inflammatory agents into circulation, leading to potential effects on blood–brain barrier integrity as well as symptoms like fatigue, hypersomnia, cognitive difficulties, or chronic inflammation that persists beyond antimicrobial treatments such as treating pathogens like memory deficits, depression or mood disorders—raising questions regarding the involvement of immunological factors in different neurological diseases [13].

### 2.4. The Role of the Immune System in Maintaining Neuronal Health

The immune system plays a pivotal role in protecting and maintaining neuronal health in multiple ways. One mechanism by which it affects neuronal activity is through signaling molecules known as cytokines that control immune responses and may have an effect on neuronal activity—proinflammatory cytokines such as IL-1, IL-6, and TNFα are believed to trigger fever responses during infections by increasing body temperature to counter infection symptoms [14].

Immune cells become activated upon contact with infectious agents, producing proinflammatory cytokines, which then induce the production and release of prostaglandins in the brain—specifically PGE_2_. PGE_2_ plays an essential role here, acting on thermosensitive neurons in the hypothalamus to induce fever; specifically, its effect is known to activate thermosensitive neurons within this area and induce fever by inducing thermosensitivity responses from thermosensitive neurons. This then triggers further heat production via thermosensitive neurons within the liver, followed by a sustained phase by brain astrocyte production of IL-6, which further stimulates PGE_2_ production; all this action provides a sustained phase fever response [15].

Cytokines play a pivotal role in activating the hypothalamic–pituitary–adrenal (HPA) axis. When immune cells release cytokines in response to pathogens, PGE_2_ is produced within brain vasculature and interacts with catecholaminergic neurons before projecting onto corticotropin-releasing hormone-containing neurons of the hypothalamus; these send projections onto corticotropin-releasing hormone (CRH), leading to elevated levels of ACTH and corticosterone. Additionally, cytokines directly influence the pituitary gland to increase the release of ACTH. This complex process demonstrates just how powerfully these proteins exert diverse effects upon this part of our immune response as well as stress regulation mechanisms [16].

Longer exposure to cytokines may result in glucocorticoid resistance, making the body less responsive to their effects and diminishing their effects on homeostasis and stress responses. Resistance predominantly manifests itself at the level of the hippocampus and impairs regulation of the HPA axis, consequently compromising one’s ability to effectively respond to stressors and maintain homeostasis [17].

Communication between the immune and nervous systems, enabled by cytokines, plays an integral part in protecting neuronal health and orchestrating physiological responses during infections or inflammation events. A thorough understanding of these complex pathways is vital to unlocking their underlying mechanisms as well as their profound implications for overall health and disease development; such knowledge could pave the way for novel therapeutic interventions aimed at maintaining harmony between these vital systems for increased human wellbeing [14].

## 3. The Neuroimmunology of Multiple Sclerosis

### 3.1. Pathophysiology of MS: Neuroimmunological Perspective

Multiple sclerosis (MS) is a devastating autoimmune condition affecting the central nervous system (CNS), comprising both the brain and spinal cord. MS is caused by immune attacks targeting myelin sheaths encasing nerve fibers axons in the CNS. These attacks cause inflammation, demyelination (depleted myelin levels), and damage to nerve fibers encased within it, resulting in inflammation, demyelination (depletion of myelin), and damage [18].

MS is a debilitating disease, targeting not only white matter axons covered in myelin but also nerve cell bodies in the gray matter of the brain and optic nerves to transmit information between the eye and brain. Over time, MS can lead to cortical atrophy, which shrinks the outermost layer of the cerebral cortex.

Individuals living with MS often experience various symptoms based on the intensity, location, and extent of inflammation, as well as the plaques’ distribution across specific body areas, including the brain stem, cerebellum (associated with balance and coordination), spinal cord, optic nerves, and white matter surrounding brain ventricles (fluid-filled cavities). The variations in plaque distribution contribute to various degrees of symptoms being experienced by MS patients [5].

Research indicates a potential link between disrupted energy metabolism in the CNS of MS patients. Higher blood pyruvate levels during fasting and after meals in MS patients experiencing relapses had been found, as well as a potential flaw in pyruvate metabolism in MS. Other researchers have also noted elevated fasting pyruvate levels in MS. However, some studies showed normal fasting lactate levels or elevated levels in only a few MS patients. An unusual increase in blood pyruvate after glucose consumption has been reported. These findings point towards potential irregularities in pyruvate metabolism in MS patients. Moreover, the rise in Krebs cycle acids, such as alpha-ketoglutarate when fasting and citrate after glucose consumption, in MS patients reinforces the idea of disrupted pyruvate metabolism being linked to MS progression. Increased pyruvate and α-ketoglutarate levels in MS were also observed. Elevated enzyme activity, including enolase, pyruvate kinase, lactate dehydrogenase (Ldh), and aldolase in the CSF of those with disseminated sclerosis, suggests they could be markers of active demyelination.

### 3.2. The Role of the Immune System in MS

#### 3.2.1. The Blood–Brain Barrier

One striking characteristic of MS lesions is the disruption of the blood–brain barrier (BBB), making its understanding a key aspect of understanding its pathogenesis [19]. The BBB serves as both a functional and anatomical separation between blood flow in the central nervous system (CNS) and neurons located outside. It consists of the vascular wall, CNS astrocytes covered with glia limitans covering them, and the perivascular space between [20]. Furthermore, its functioning provides many essential functions essential for proper brain function, such as the regulation of proper ionic concentrations.

**Figure 1:**
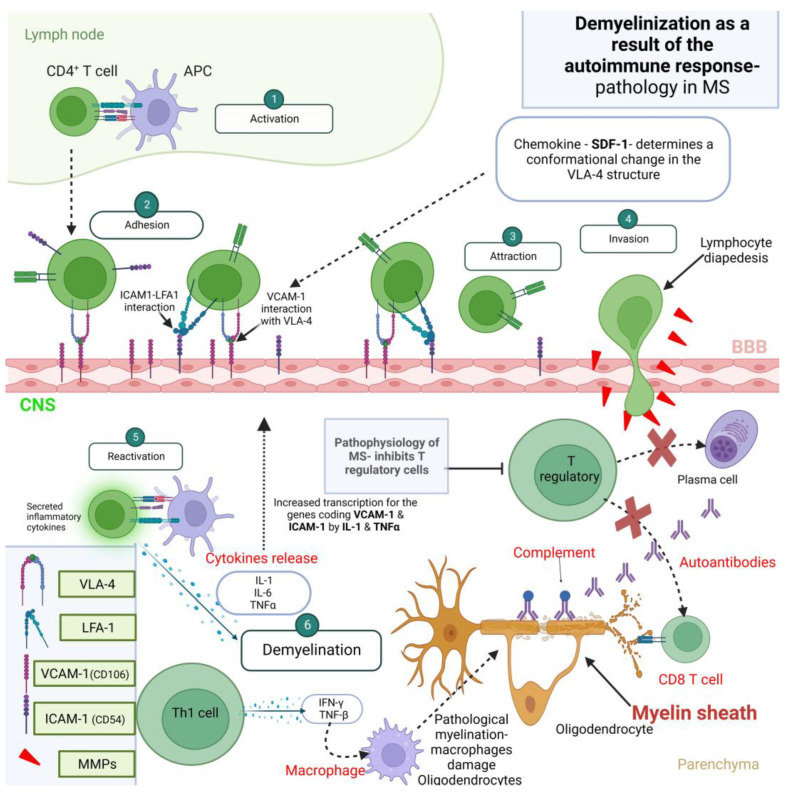
The immunopathogenic model of multiple sclerosis (MS) posits that a confluence of genetic and environmental variables, including viral infections, bacterial exposure, and superantigens, synergistically contribute to the heightened activation of myelin-reactive T cells within the circulatory system of individuals afflicted with MS. This phenomenon is further exacerbated by the elevated expression of endothelial adhesion molecules, namely intercellular adhesion molecule-1 (ICAM-1) and vascular cell adhesion molecule-1 (VCAM-1), which facilitate the translocation of T cells into the central nervous system (CNS). The motility of these T cells is augmented by chemokines and the synthesis of matrix metalloproteinases, which enzymatically degrade extracellular matrix proteins to smooth the migration pathway. This collectively culminates in the T cells’ successful transgression of the blood–brain barrier (BBB). Upon entry into the CNS, T cells become activated via interactions with antigen-presenting cells, initiating the release of pro-inflammatory and cytotoxic mediators. These substances inflict tissue damage and compromise the integrity of the myelin sheath through several mechanisms, including cytokine-mediated injury, antibody-mediated phagocytosis of myelin antigens by macrophages, complement system activation, and direct cellular damage mediated by CD4+ and CD8+ T cells. Regulatory changes at the transcriptional level, instigated by cytokines such as tumor necrosis factor-alpha (TNF-α) and interleukin-1 (IL-1), lead to the upregulation of VCAM-1 in endothelial cells. Notably, the integrin very late antigen-4 (VLA-4) does not bind to its designated ligands, including VCAM-1 and fibronectin, unless activated by chemotactic agents or additional stimuli, often synthesized by endothelial cells or other cellular entities at the site of inflammation. One such activating chemokine is stromal cell-derived factor-1 (SDF-1). ICAM-1, a type of intercellular adhesion molecule, is constitutively present in nominal concentrations on leukocytes and endothelial cells. In response to cytokine stimulation, however, these concentrations markedly escalate. ICAM-1 serves as a ligand for lymphocyte function-associated antigen-1 (LFA-1), an integrin present in leukocytes. LFA-1 plays a pivotal role in leukocyte emigration, facilitating their egress from the bloodstream and entry into tissues. Moreover, LFA-1 partakes in the processes of cytotoxic T-cell-mediated and antibody-mediated cellular destruction, enacted by granulocytes and monocytes. The mechanism governing T-lymphocyte infiltration into the CNS is explicable via the elevated expression of adhesion molecules on the endothelial cells constituting the BBB in MS cases. This assertion is further substantiated by the observation of these molecules on inflammatory cells such as macrophages and lymphocytes in MS lesions. It is a widely accepted notion that cytokines released by Th1 cells, including interferon-gamma (IFN-γ) and TNF-beta (TNF-β), are capable of macrophage activation, subsequently causing damage to oligodendrocytes and leading to pathologic alterations in myelination.

While the term “barrier” might connote something static, the blood–brain barrier (BBB) is actually dynamic, providing processes like immunological surveillance. Lesions occur when leukocytes migrate into the brain, causing inflammation. This process comprises two steps, starting with initial migration across postcapillary venules into the perivascular space, followed by passage through the glia limitans to brain parenchyma [20]. The perivascular space provides monocytes with an area for normal immunosurveillance while also serving as lymphatic drainage. Furthermore, animal models show two additional routes through which leukocytes enter the CNS. Understanding these pathways provides insight into the workings of the blood–cerebrospinal fluid (CSF) barrier (BBB), including its function in immune cell infiltration during MS (Figure 1).

#### 3.2.2. The Role of T Cells

T cells play an essential role in the pathogenesis of multiple sclerosis (MS), as evidenced by EAE animal models and HLA class II genes that strongly associate with MS. EAE, which mimics MS in animals, can be caused by immunizing rodents with myelin peptides, leading to an immune response targeting myelin in the central nervous system (CNS). Initial investigations suggested that auto-reactive CD4+ T cells producing interferon-gamma (IFN-γ) were the main contributors to inflammation in MS lesions; however, more recent investigations have thrown other cell types, particularly T helper 17 (Th17) cells, into focus as primary aggressors of MS lesions. These Th17 cells are responsible for producing pro-inflammatory cytokines such as IL-17 and IL-6, while their activity is governed by IL-23. These findings demonstrate the complex immune response involved in MS pathogenesis as well as Th17 cells’ participation in driving inflammation processes [21].

CD4+ Th17 cells play an essential part in the development of EAE. MS lesions exhibit both CD4+ and CD8+ cells, which express IL-17. Th17 cells use the CCR6 chemokine receptor to travel from the choroid plexus into the cerebrospinal fluid (CSF) and perivascular spaces, inducing inflammation and damaging neurons and glial cells along their path. Th17 cells play a pivotal role in EAE and MS pathogenesis by secreting GM-CSF, exacerbating an already intense inflammatory response. Furthermore, they break through the blood–brain barrier (BBB) with their release of IL-17 and IL-22, breaking it down further by drawing in immune cells into the CNS through disruption of BBB. These findings demonstrate their crucial contribution to both diseases’ pathologies, contributing to inflammation and damage seen within MS lesions [22].

Chemokines play an essential role in recruiting immune cells across the blood–brain barrier (BBB) during an episode of inflammation. MS lesions involve specific endothelial-wall-expressed chemokines interacting with receptors present on T cells to enable their extravasation into the CNS. Our research focused on investigating the expression of different chemokine receptors on T-cell clones derived from both blood and cerebrospinal fluid of an MS patient treated with glatiramer acetate (GA). CCR_4_, CCR_5_, CCR_6_, and CXCR_3_ emerged as particularly pertinent receptors to investigate. Their activation coincided with T-cell migration patterns that corresponded with specific receptor expression patterns, suggesting these receptors might play an essential role in migration and infiltration into the CNS during MS inflammation [23].

Based on this study’s findings, reduced glatiramer acetate (GA) efficacy may be attributable to inflammation and early activation of GA-reactive cells. A better understanding of T-cell migration into the central nervous system (CNS) holds great promise in potentially increasing GA’s effectiveness and ultimately bettering treatment outcomes for MS patients. By exploring mechanisms governing T-cell infiltration into CNS, we may open doors for more targeted and efficient therapies that benefit individuals living with MS [24]. 

#### 3.2.3. The Role of B Cells

Immunoglobulin G1, present in most diagnosed multiple sclerosis (MS) patients’ cerebrospinal fluid (CSF), may point to B cells as potential agents in its pathogenesis. Myelin-reactive antibodies have been detected, yet their exact significance remains uncertain, similar to antigens’ significance. Immunoglobulin and complement are present in lesions associated with MS, suggesting their possible pathogenicity. Additionally, in patients at later stages of MS, B lymphoid follicles, T cells, and antigen-presenting cells have been detected in the meninges, suggesting potential contributions by B cells through antigen presentation, cell interactions, or immunoglobulin production by plasma cells. However, it must be kept in mind that B-cell activity in MS could be a result of an autoimmune reaction and not be the sole trigger of multiple sclerosis. More research needs to be conducted in order to fully comprehend how B cells play into multiple sclerosis pathogenesis [25].

Experimental animal models like EAE have well-documented antigens responsible for disease, such as myelin proteins. Unfortunately, for humans with multiple sclerosis (MS), specific triggers that trigger this illness remain undecided despite numerous research efforts examining various candidate antigens, but none have emerged as definitive culprits [26].

Due to similarities between EAE and MS, myelin or myelin-derived peptides were initially considered promising candidates as potential causal agents. However, responses to antigens have proven nonspecific, suggesting the involvement of multiple antigens or epitopes that spread once the disease begins. One recently proposed antigen is ab-crystallin, an antibody-binding protein not naturally found in human myelin but observed in early active MS lesions. Patients diagnosed with MS possess antibodies against ab-crystallin in their cerebrospinal fluid (CSF). Studies on mice demonstrated that knocking out its gene caused more intense EAE with elevated cytokine levels, suggesting its protective role has been compromised by an autoimmune response that disrupted it [27]. 

In an animal model of MS, antibodies targeting neurofascin cause axonal damage, disrupt neuronal conduction, and worsen the disease. Neurofascin is a cell adhesion molecule found on the surface of neurons. It plays a critical role in the formation and maintenance of nodes of Ranvier, which are essential for the rapid conduction of nerve impulses. Any disruption to these nodes can impair nerve signaling, leading to neurological symptoms. For an autoantibody in the bloodstream to damage the CNS, it must first cross the blood–brain barrier (BBB) and then bind to its target within the CNS. In MS, both conditions are met. The disease increases the BBB’s permeability, allowing antibodies to enter the CNS. Moreover, these antibodies can recognize and bind to the native structure of NF186, a type of neurofascin. These antibodies can bind to NF186 in vivo, potentially intensifying axonal damage in MS patients, especially those with high antibody levels. While 20–30% of MS patients have high levels of these antibodies, they are also found in some healthy individuals. This is not surprising, as similar patterns are seen with other autoimmune responses in MS. In conclusion, we have identified neurofascin as a potential target in some MS patients. Antibodies against neurofascin can cause axonal damage in CNS inflammatory diseases, suggesting potential new therapeutic avenues.

Neurofascin, another candidate antigen expressed on neuronal axons and linked with MS, may play a part in contributing to axonal damage. The presence of antibodies against neurofascin in MS patients suggests such damage may contribute to MS pathogenesis and treatment interventions. Even so, further research must continue in this field to understand the antigenic targets for MS disease pathogenesis and potential therapeutic interventions more deeply [28].

#### 3.2.4. The Role of NK Cells

NK cells are large granular lymphocytes with the capacity to spontaneously eliminate target cells without prior sensitization, as well as serving a regulatory function by secreting cytokines and engaging in cell-to-cell interactions. Functionally, natural killer (NK) cells play an essential role in initiating immune responses against viral infections and slowing tumor growth. Their actions are tightly regulated through activating and inhibitory receptors, which guide their responses to stimuli. NK cells play an essential role in our immune system’s defense against infections and cancerous growth by identifying stress-induced ligands on target cells using specific receptors like natural cytotoxicity receptors and C-type lectin receptor NKG2D. Through their unique capabilities, they serve as a frontline defense against infections or cancerous growth [29].

NK cells’ role in multiple sclerosis (MS) remains an area of intensive investigation, with both positive and negative correlations observed in studies. Studies with an experimental animal model of MS (EAE) indicate possible protective benefits from their depletion in terms of more severe EAE symptoms and increased CNS pathology due to CD4+ T cells being killed directly by them; however, conflicting results have also been reported showing both beneficial and detrimental impacts from their involvement with EAE [26].

Functional activity of NK cells tends to be lower among MS patients compared with healthy individuals, leading to decreased numbers in their blood. Lower numbers have been linked to higher risks of relapse and lesion formation for MS patients. While its exact role in MS pathogenesis remains complex and multifaceted, evidence indicates NK cells could exert a major impact on disease outcomes. Further investigation will help shed more light on this intricate relationship between immune response mechanisms and NK cells, potentially providing insights into new therapeutic strategies and approaches for managing MS [30].

**Figure 2:**
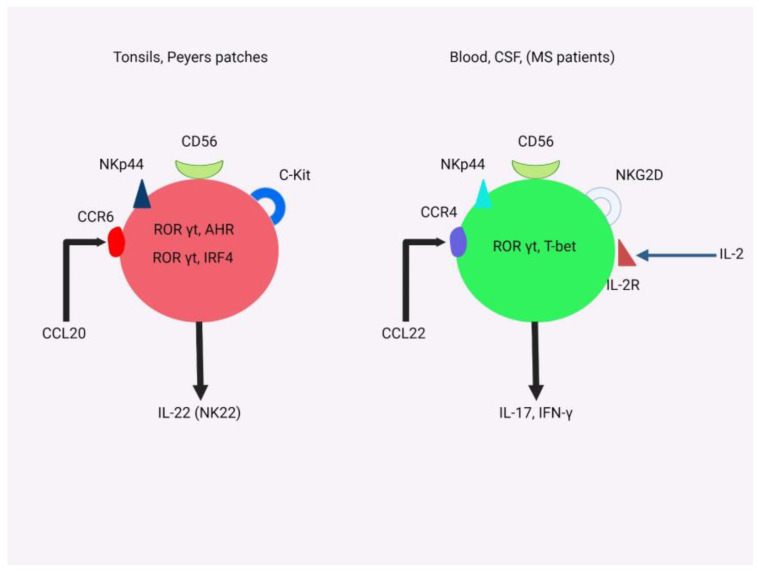
Comparison between NK22 cells and NK17/NK1 cells. NK22 cells are primarily located in tissues such as tonsils and Peyer’s patches, while NK17/NK1 cells originate from human blood when activated in vitro with IL-2 and are notably present in the CSF of MS patients even in the absence of activation. NK22 cells predominantly express the NK cytotoxicity receptor NKp44. NK17/NK1 cells express receptors NKp30, NKG2D, NKp44, NKp46, and CD158, although the expression of NKp44, NKp46, and CD158 is less pronounced compared to other NK cell cytotoxicity receptors. NK22 cells express CCR6, and both secrete and respond to CCL20/MIP-3α. NK17/NK1 cells, on the other hand, express CCR4 and both secrete and respond to CCL22/MDC. NK22 cells express transcription factors RORγ, AHR, RORα, and IRF4. NK17/NK1 cells express RORγ and T-bet, leading them to secrete both IL-17 and IFN-γ.

Researchers have suggested that interactions between NK cells and dendritic cells (DCs) could play an integral role in understanding their impact on MS (Figure 2). Studies indicate that GA (glatiramer acetate), an effective MS treatment medication, can alter these communications between NK cells and DCs to decrease autoantigenicity presentation to autoreactive T cells and thus lessen inflammation [31].

Studies conducted with mice with experimental autoimmune encephalomyelitis (EAE), an animal model of MS treated with GA, support the concept that GA enhances natural killer cell (NK cell) cytotoxicity. Furthermore, in MS patients undergoing GA treatment, their NK cells demonstrated significantly enhanced antitumor and DC killing abilities when compared to their pre-treatment levels, suggesting interactions between NK cells and DCs as potentially being key pathways underlying its effects in MS management and meriting further exploration to better understand how this drug acts when treating this disease [32]. 

Multiple sclerosis (MS) involves many complex interactions between NK cells and MS, which may be both positive and negative in nature. To fully grasp their impact, further research must be conducted, particularly to understand the specific effects of various subsets of NK cells in relation to MS development and response to treatments; such deeper knowledge will reveal insights into potential therapeutic strategies or interventions that may help manage MS more effectively.

## 4. Immune Mechanisms in Neurological Disorders

### 4.1. Immune-Related Neurological Disorders: MS and Beyond

Autoimmune disorders such as multiple sclerosis (MS) involve the immune system directly attacking body cells. In MS, immune cells target and attack the myelin sheath surrounding axons to cause demyelination and disability. Historically, this disease was classified as either relapsing-remitting or progressive; now, however, these classifications overlap, creating a spectrum of disease manifestations [33].

Relapsing symptoms in MS are associated with acute inflammation, often due to the infiltration of immune cells from the periphery into the central nervous system (CNS). Progressive symptoms, however, tend to be caused by neuroinflammation. Immunotherapy has proven moderately successful at treating MS relapsing symptoms; however, due to targeting peripheral immune system components only, it thus fails to address progressive symptoms. Immunotherapy’s success at treating relapsing symptoms underscores how important immune interactions between central and peripheral systems are in MS onset and progression, opening potential avenues for improving therapeutic targeting for progressive MS cases [34].

Although much about MS’s etiology remains unknown, experimental autoimmune encephalomyelitis (EAE) models have become an invaluable way of studying this illness and uncovering potential mechanisms. A typical one involves CD4+ T cells migrating into the CNS. These infiltrations may result from disruptions in either the blood–brain barrier (BBB) or blood–cerebrospinal fluid barrier (BCSFB) and may include T cells that recognize host myelin as antigen during infiltration or become activated later. These reactive T cells release proinflammatory cytokines that trigger microglia activation and attract peripheral macrophages that contribute to myelin destruction. Microglia become activated when they release proinflammatory cytokines, exacerbating neuroinflammation further and disrupting the BBB, leading to greater infiltration of peripheral immune cells into the central nervous system. Neuroinflammation caused by microglia and astrocytes persists throughout disease progression as neurons undergo demyelination and degeneration, which makes understanding these complex mechanisms essential to progressing knowledge about MS and developing more effective treatment approaches [35].

Microglia were once thought to be solely pro-inflammatory cells; however, recent discoveries show they also play an anti-infiltrative function by restricting macrophage infiltration into the central nervous system (CNS). Studies have also uncovered activation phenotypes resembling “disease-associated microglia”, suggesting similarities with other neurodegenerative conditions. Furthermore, their interaction is essential in multiple sclerosis development and progression and presents an attractive target for therapeutic interventions [36].

Neutrophils have emerged as key players in the severity of multiple sclerosis (MS), as their count correlates directly to disease activity. Studies using MS models have also demonstrated that depleting neutrophils leads to reduced disease severity, suggesting their potentially detrimental role in an autoimmune disorder like MS. CXCR_2_ signaling might play a part in mediating neutrophil-induced damage and its interactions with microglia, providing a promising therapeutic target for managing MS [37].

So, MS research highlights the critical role played by immune cell interactions in the development of multiple sclerosis (MS). Recognizing and targeting these interactions may provide effective therapies to impede or reverse MS progression and lead to better patient outcomes.

### 4.2. The Role of Neuroimmunity in the Pathogenesis of Neurological Disorders

Dysregulated immune responses in the central nervous system (CNS) play an essential part in inducing neuroinflammation and contributing to various neurological conditions, such as Alzheimer’s, Parkinson’s, multiple sclerosis, and stroke. Microglia cells secrete inflammatory proteins that cause neuronal damage while worsening symptoms, and understanding neuroimmunity offers valuable insights into potential therapeutic targets for these complex neurological illnesses [35].

## 5. Clinical Neuroimmunology Research

### 5.1. Recent Advancements in Clinical Research of Neuroimmunological Disorders

Recent advances in neuroimmunology research have made substantial strides, providing unprecedented insights into the underlying mechanisms, diagnosis, and treatment of neurological conditions involving the immune system. Advancements in technology, including sophisticated imaging techniques such as MRI and PET scans, as well as high-throughput genomics and proteomics analyses, have enabled more in-depth investigation of the relationship between nervous and immune systems. PET is a potent functional imaging technique that can explore both healthy and affected brains. It offers a non-invasive way to measure specific biological markers, enhancing our comprehension of intricate central nervous system conditions like multiple sclerosis (MS). While MRI remains pivotal in tracking MS’s clinical progression, PET has traditionally played a supplementary role. Nevertheless, recent advancements in PET imaging present opportunities to examine the MS brain in ways MRI cannot. PET can delve into the root causes of neuroinflammation, neuronal issues, demyelination, and remyelination in MS. Additionally, PET’s ability to quantitatively assess molecular targets might be beneficial in future drug development clinical trials. However, the widespread adoption of PET is constrained by the significant expenses associated with cyclotrons and radiochemical labs. Discovering novel biomarkers has proven immensely helpful in early disease diagnosis and monitoring disease progression. Immunotherapies that target specific immune components have demonstrated promising results in clinical trials, revolutionizing neuroimmunological disorders management. These advances hold great promise in improving patient outcomes while furthering our understanding of these complex conditions [1].

### 5.2. Methodologies Used in Clinical Neuroimmunology Research

Clinical neuroimmunology research is an interdisciplinary field that draws upon clinical, immunological, and neurobiological methods to gain a comprehensive understanding of neuroimmunological disorders. This field conducts large-scale epidemiological studies to study their prevalence and risk factors associated with them, and advanced imaging technologies like MRI allow researchers to visualize brain and spinal cord lesions as well as track disease progression over time [38].

Immunological assays, such as flow cytometry and cytokine profiling, play an integral part in characterizing immune cell populations and their activation status in relation to neurological disorders under investigation. Genetic studies are performed in order to identify susceptibility genes as well as their influence in contributing to their development and progression [39].

Clinical trials play an integral part in assessing the safety and efficacy of various immunomodulatory and neuroprotective therapies used to treat neuroimmunological disorders in patients. Employing different research methodologies together contributes to expanding our knowledge about these complex conditions while developing interventions designed to enhance patient outcomes [40]. 

### 5.3. Challenges and Opportunities in Current Research

As clinical neuroimmunology research advances, however, it faces several difficulties. One key hurdle stems from the diverse nature of neurological conditions and associated immune responses. Due to this diversity, it becomes challenging to create universal therapeutic approaches that work for all patients [41].

Another barrier lies within the blood–brain barrier, which hinders the effective delivery of immunotherapies to the central nervous system (CNS). Finding appropriate strategies to overcome this barrier will improve treatment results and thus increase treatment efficacy. As part of clinical research, evaluating the long-term safety and efficacy of immunomodulatory treatments remains a paramount focus. A comprehensive evaluation is necessary to safeguard patient wellbeing as well as identify long-term benefits from therapies like these. Lacking validated biomarkers for disease prognosis and treatment response poses another difficulty: without reliable indicators, it becomes hard to tailor treatments specifically to individual patient needs and delay personalized medicine approaches. Ethical considerations in clinical research require careful consideration, from obtaining informed consent from patients to addressing data privacy concerns. Maintaining a balance between scientific advancement and patient welfare/privacy rights must always be prioritized for responsible research conduct [42].

### 5.4. Case Studies: Application of Clinical Research in Managing MS and Other Neurological Disorders

Clinical neuroimmunology research has had a substantial effect on the management of multiple sclerosis (MS) and other neurological conditions. Disease-modifying therapies, including interferon-beta, glatiramer acetate, monoclonal antibodies targeting B cells or T cells, and stem cell transplantation or gene therapies, have proven highly successful at reducing relapse rates and slowing progression. Early diagnosis with advanced techniques like MRI scans or cerebrospinal fluid analysis allows timely treatment initiation, leading to better long-term results, while stem cell transplantation or gene therapies show promise at slowing disease progression or even stimulating repair within MS patients [43].

Neuroimmunological conditions such as neuromyelitis optica spectrum disorder (NMOSD) and autoimmune encephalitis have seen great strides made through clinical research, with specific autoantibodies having been identified, providing targeted therapies. Rituximab, a B-cell-depleting agent, has been effective at managing NMOSD attacks while decreasing disability progression and recurrences, and corticosteroids, intravenous immunoglobulins, and plasmapheresis therapies have all proved beneficial when treating patients suffering from autoimmune encephalitis patients [44,45].

Neuroimmunology research holds the promise of further improving the management of complex neurological conditions, leading to improved patient outcomes and an enhanced quality of life.

## 6. Neuroimmunological Aspects of Health and Diseases

### 6.1. The Impact of Neuroimmunology on General Health

**Figure 3:**
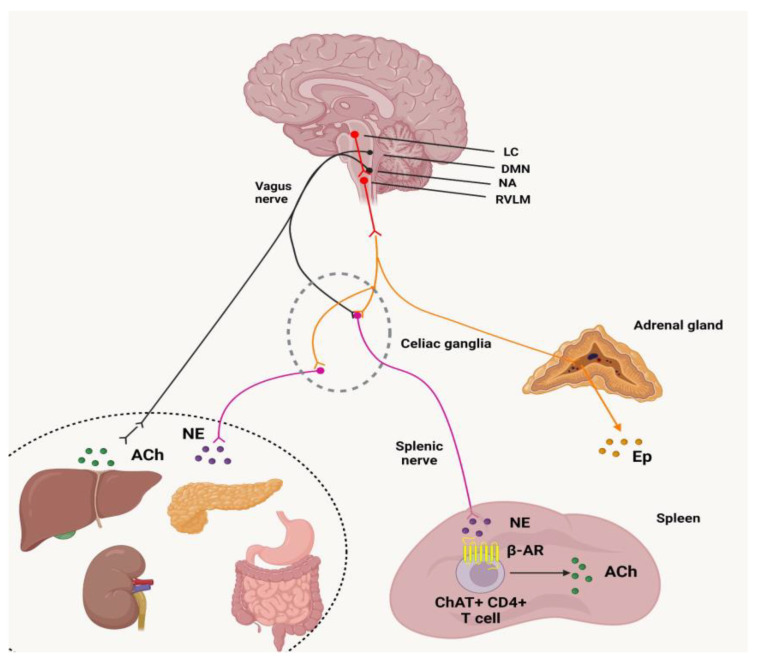
Autonomic neurons involved in regulating immune responses. Neurons of the efferent vagus nerve have their origins in the dorsal motor nucleus of the vagus (DMN) and the nucleus ambiguus (NA) located within the brainstem’s medulla oblongata. These initial cholinergic neurons extend to various visceral organs in the thoracic and abdominal regions, encompassing the lungs, heart, liver, gastrointestinal system, kidneys, and pancreas. They engage with postganglionic vagal neurons either close to or inside the organs they innervate, with these neurons primarily releasing acetylcholine. The preganglionic neurons of the vagus nerve also end at the celiac ganglia and the superior mesenteric ganglion, which is where the splenic nerve begins.

The Global Burden of Diseases (GBD) includes various non-communicable conditions like cardiovascular diseases, cancer, and chronic obstructive pulmonary disease. All three share risk factors like smoking and diet as well as pathophysiological causes like oxidative stress, inflammation, and excessive sympathetic activity [46]. This article proposes an innovative solution to predict, understand, prevent, and potentially treat these illnesses by employing neuroimmunology with vagal neuro-modulation as its foundation (Figure 3).

Vagal nerve activity plays an integral part in controlling frontal brain activity, which in turn influences unhealthy lifestyle behaviors. The aim of this article is to introduce this paradigm to medicine and public health practitioners while emphasizing its significance for disease prevention and management. Epidemiological evidence shows that increased vagal activity, as measured by greater heart rate variability (HRV), independently predicts decreased GBD risk as well as better outcomes in those already living with GBD conditions [47].

Neuroimmunology is the study of how the nervous and immune systems interact, playing key roles in neural development, homeostasis, plasticity, and behavior modification [1]. While its potential benefits for human health and the treatment of neurological and psychiatric disorders are evident, translating research findings to clinical applications remains challenging due to knowledge gaps, optimal intervention timing issues, and a lack of tools that enable visualization or modulation.

As a way of meeting these challenges and pushing neuroimmunology forward, we propose ten research questions that, if explored in depth, could yield tangible advances over the short to medium term. These questions cover various aspects of neuroimmune interactions and their implication for health, disease, and various stages of life. As key themes in answering each question, we emphasize four cross-cutting themes critical to effective investigation: (i) understanding the two-way interactions of neuroimmune interactions; (ii) considering biological context (healthy state, disease state, and lifespan aspects); (iii) employing appropriate tools and technologies for better understanding underlying mechanisms; and (iv) translating research findings into practical clinical applications [48].

Though these questions do not encompass every knowledge gap in neuroimmunology, they address areas with immediate and broad impacts. Our goal in outlining research priorities is to foster collaboration among current and future research teams, working cross-disciplinarily towards furthering neuroimmunology for significant advancements in human health and disease treatment.

### 6.2. The Role of Neuroimmunology in Non-Neurological Diseases

The brain has long been considered immunologically unique due to the limited immune reaction seen here compared to peripheral tissues. Early studies demonstrated that transplanted foreign tissues survive longer after transplanting them into the brain than elsewhere, which suggests some degree of immune privilege [49,50]. One theory behind this phenomenon attributed this to the blood–brain barrier (BBB), which was thought to prevent immune cells and molecules from entering from the bloodstream into the brain via lymphatic vessels in its vicinity—further supporting T-cell immunity’s surveillance by T cells.

Recent evidence is increasingly challenging this assumption and suggests that T cells do visit the brain. Although the BBB provides protection from passive protein entry but not from active cell entry, studies suggest drainage of cerebrospinal fluid and intracerebrally injected antigens to deep cervical lymph nodes is used as a connection between brain immune systems and peripheral ones [51,52].

Becher et al. (2000) illustrate the immunoreactivity of the brain as being in an active state. Thus, attention has shifted towards creating and maintaining an immunosuppressive microenvironment through factors like TGFβ, prostaglandins, and neurotrophins that are released by astrocytes and neurons releasing TGFβ, prostaglandins, and neurotrophins which serve to suppress immune responses while mitigating excess inflammation within the brain [53]. 

B cells play an integral part in immune responses, both positively and negatively regulating them. B cells have been associated with neuroimmunological diseases like myasthenia gravis (MG), multiple sclerosis (MS), Guillain–Barré syndrome (GBS), and Lambert–Eaton myasthenic syndrome (LEMS), with B cells playing a part in their pathogenesis by producing antigen-specific antibodies which stimulate T-cell activation and cytokine production while contributing to optimal T-cell activation and production by T cells [54]. 

Though research on B cells and autoantibodies in neuroimmunological diseases remains relatively scarce, ongoing investigations in this field continue to shed light on their pathogenic roles and gradual involvement. Understanding how B cells contribute to disease development and progression is key for advancements in this field, with promising clinical applications and greater insight into underlying causes for neuroimmunological conditions expected as B-cell research expands further [55]. Eventually, it is hoped that further insight from research on neuroimmunological conditions can lead to improved treatments or management methods.

### 6.3. Neuroimmunological Perspectives in Chronic Diseases

An experiment was carried out in order to determine the presence of T cells in the brain parenchyma of individuals with Alzheimer’s disease (AD), other degenerative dementias, and control subjects using semi-quantitative analysis of immunohistochemically stained tissue sections. T cells were present in all cases examined; notably, more T cells could be observed in AD cases than in any other cases studied [56].

T cells found in AD patients’ brains appeared activated but not fully differentiated, without evidence of antigen-triggered clonal expansion. Instead, local inflammation may explain this pattern of T-cell accumulation and activation within AD brain tissue. Our findings shed light on their possible involvement in AD pathology as well as suggest a possible role inflammation plays in driving T-cell activity within this setting; further investigation should elucidate their exact mechanisms and implications in Alzheimer’s disease [57].

Autoimmunity is a significant contributor to diseases affecting both the central and peripheral nervous systems. The causes and clinical manifestations of these conditions vary considerably. Among the key causes is dysregulated complement activation at specific sites—this has been implicated as one cause in various neurological conditions like Guillain–Barré syndrome and neuromyelitis optica, where autoantibodies trigger activation of the complement system and damage self-tissues. Understanding this interaction is integral for understanding the pathogenesis behind these neurological diseases [58].

Neuroimmunological diseases and their treatments pose challenges to the immune system, elevating the risk of infections and severe illnesses. Consequently, vaccinations play a crucial role in the clinical management of these conditions. However, the diverse array of immunotherapies utilized in treating neuroimmunological diseases, especially multiple sclerosis and neuromyelitis optica spectrum disorders, can also impact immune responses to vaccines [59]. 

Polyspecific intrathecal immune response (PSIIR), commonly referred to as the “MRZ Reaction” (M = measles, R = rubella, and Z = zoster; optionally herpes simplex virus, HSV), refers to intrathecal immunoglobulin synthesis against two or more unrelated viruses found in cerebrospinal fluid [60]. While widely recognized as an indicator for multiple sclerosis (MS), PSIIR, an autoimmune–inflammatory neurological disease typically affecting early adulthood with widespread damage throughout its central nervous system (CNS), remains incompletely understood across other CAINDs that show positive results. 

### 6.4. The Influence of Environmental and Lifestyle Factors on Neuroimmune Health

Behavioral neuroscience has been profoundly shaped by the understanding that brain development occurs throughout adolescence and adulthood. Studies over time have uncovered extensive research that indicates that adolescence is characterized by temporary differences, leading to greater risks, rewards, and susceptibility for affective disorders (as discussed here and further below). Importantly, behaviors and mental health disorders do not arise solely from neuronal activity in the brain itself. Instead, communication occurs between peripheral factors like immunity. Neuronal and immune interactions play a pivotal role in regulating cognitive and behavioral functioning as well as any dysfunctions throughout life, so for an accurate understanding of adolescent brain development, we propose conducting in-depth developmental investigations of both peripheral and central immune mechanisms [61].

Due to the devastating impacts of substance use disorders on health and wellbeing, extensive research has been carried out to uncover factors contributing to their progression from initial drug use to pathological drug use. Pinpointing these risk factors is essential in creating strategies for mitigating risks and decreasing prevalence rates among the population. We will review emerging research that explores interactions among peripheral immune system elements, gut microbiome composition, and central nervous system functions in driving pathological drug use [62].

Multiple sclerosis (MS) is a chronic, progressive, inflammatory, and degenerative condition that impacts millions of people worldwide, mostly impacting the central nervous system. The interplay among glial, neural, and immune cells plays an integral part in driving MS pathology, providing opportunities for therapeutic interventions [63]. We explore contributing risk factors behind MS development, current treatments available to modify it, and potential emerging technologies to fill clinical gaps, as well as identify novel avenues of therapy targets in this review article. 

MS displays gender disparities in both immune reactions and neurodegeneration, influencing disease vulnerability and its progression. Interestingly, while women seem more prone to MS due to immune system differences, these differences do not align with those in the CNS. This raises the following query: if women have a higher disease incidence and stronger peripheral immune responses, why do they not experience quicker disability progression? In fact, men seem to progress faster. We theorize that gender-specific factors might have distinct impacts on the immune system compared to the CNS, leading to differences in disease susceptibility versus the rate of disability progression.

## 7. Developments in the Immunotherapy of Neurological Disorders

### 7.1. Current Approaches to Immunotherapy in Neurological Disorders

Imaging plays an integral role in diagnosing brain metastases, with MRI being the preferred imaging technique due to its greater sensitivity compared to CT. It effectively reveals the size, number, and distribution of central nervous system (CNS) lesions, such as solid or pseudospherical lesions. Brain metastases typically appear at grey–white junctions, with cerebral hemispheres being most prevalent (80%), followed by the cerebellum (15%) and brainstem (5%) [64]. Certain primary malignancies like melanomas, choriocarcinomas, germ cell tumors, thyroid cancers, or renal cell carcinomas are more susceptible than others to hemorrhage-related brain metastases.

Postoperative MRI can help distinguish between residual tumor material and blood byproducts after surgical removal of CNS lesions, according to data from malignant glioma studies. Changes in tumor diameter after stereotactic radiosurgery (SRS) can vary widely: approximately one-third of lesions experience transient increases in volume that usually stabilize or return to their original size over time [65].

Immunotherapy holds promise for treating neurological diseases, similar to its successes in treating cancer and autoimmune conditions. By harnessing immune responses, along with their capacity for tissue repair support, immunotherapy may hold great promise as an approach [66].

Multiple sclerosis (MS) was once treated by treating symptoms alone; however, thanks to recent advancements in our understanding of MS’s underlying mechanisms, treatment options are expanding quickly in an attempt to slow or stop its progression. Immunological therapies have proven particularly successful at this, offering hope of better disease control before severe neurological disabilities manifest themselves. Ongoing research capitalizes on the advances made in immunoregulation understanding by offering more targeted and selective immunological treatments targeting myelin antigens while decreasing side effects [67].

### 7.2. Advances in MS Immunotherapy

Multiple sclerosis (MS) remains unknown with certainty, although evidence points towards a role for the immune system in its progression. Genetic and environmental risk factors both play a part in susceptibility [68]. Researchers have extensively researched experimental autoimmune encephalomyelitis as an animal model for MS that displays T-cell-mediated inflammation within the central nervous system that often results in demyelination or nerve fiber damage. 

Multiple sclerosis (MS) immunotherapy can lead to numerous side effects, which could impact treatment decisions based on an analysis of risks and benefits. One such adverse reaction is progressive multifocal leukoencephalopathy (PML), most frequently seen after taking natalizumab. Factors such as prior immunosuppressive therapy, presence of John Cunningham virus (JCV) antibodies, and treatment duration exceeding two years increase the risk of natalizumab-associated PML. Its incidence among MS patients treated with all three risk factors is estimated at 13 cases for every 1000 cases treated with this medicine. PML cases have also been associated with treatments of fingolimod and dimethylfumarate, leading to significant considerations as to whether early intervention with potent anti-inflammatory drugs such as fingolimod and dimethylfumarate is the optimal approach to prevent long-term disability. There has been considerable debate regarding which sequence and combination therapy would best address long-term disability, and ethical considerations related to placebo-controlled trials for relapsing MS have further complicated matters where withholding treatment is seen as unethical [69].

Current understandings of multiple sclerosis (MS) as an autoimmune disease have been informed by research on MS lesions and animal models, especially experimental autoimmune encephalomyelitis (EAE) [70]. Such studies not only provided key insight into potential mechanisms but also into fundamental immunological processes.

This article is divided into two sections. The first addresses essential immunological concepts and offers an imagined scenario for the immunopathogenesis of multiple sclerosis (MS). The second provides a critical review of various biotech-based immunotherapies with particular attention paid to their immunological principles, clinical evidence, and potential challenges.

One promising approach involves coupling intact proteins or multiple myelin-derived peptides to single cells like splenocytes or erythrocytes to enable simultaneous targeting of various T-cell specificities. This concept is especially significant for MS antigen-specific immunotherapy as tolerance of multiple T-cell epitopes is believed to be essential in effective disease treatment due to epitope spreading [71].

Altered peptide ligands (APLs) are modified versions of antigenic peptides that can switch their response from being an agonist to antagonist by disrupting specific hydrogen bond interactions with T-cell receptors. This has been shown to reduce Th1 responses significantly. When conjugated to reduced mannan, this cyclic APL induces significant Th1 reduction and moderate Th2 responses [72].

Another study using APLs of linear and cyclic MBP83-99 analogs, MBP83-99(A91, A96), conjugated to reduced mannan, showed an ability to redirect immune responses away from Th1 towards Th2 [73]. Thus, using reduced mannan as an immunotherapy strategy offers potential immunotherapeutic solutions for MS patients.

### 7.3. Challenges in Developing Effective Immunotherapies

Recent clinical research in multiple sclerosis (MS) has provided valuable insights into treating clinically isolated syndromes and secondary progression, as well as the effectiveness of different immunomodulatory therapies—findings with significant implications for optimizing the care of MS patients [74].

At present, treatment of autoimmune diseases typically includes corticosteroids and immunosuppressive therapies. More refined approaches have also been incorporated, such as interferon β (IFN-β) and glatiramer acetate (GA), and both medications have been employed successfully in managing MS [75].

However, despite progress made in MS research, significant challenges still exist. Progressive MS remains untreatable, and efforts to repair injured axons and protect neurons are limited [76]. Recent advances have laid the foundation for developing targeted immunotherapies based on genetic and genomic discoveries, hopefully expanding patient therapeutic options.

MS is characterized by an adaptive immune response involving regulatory T cells (Tregs), with functional deficiencies being seen within this network of Tregs being linked with disease activity and progression. Thus, inducing Tregs as part of MS immunotherapy should be one key goal [77].

Since 1993, when interferons became available as treatments for MS, significant progress has been made in immunotherapy for MS. More specific immunomodulatory drugs have become available, showing increased efficacy tailored to each person with MS.

Targeting immune elements involved in the immunologic cascade is one approach to treating MS, immunoactive drugs that focus on either the innate or adaptive immune response have been developed. We discuss humoral-targeted immunotherapies for MS, such as Rituximab, Ocrelizumab, and Ofatumumab, that show promise as B-cell-depleting agents—some agents, such as Atacicept, were discontinued due to increased inflammation activity compared with the placebo [78].

### 7.4. Future Directions in Immunotherapeutic Strategies for Neurological Disorders

Precision oncology and immunotherapy hold great promise in developing more effective, well-tolerated therapies against highly aggressive forms of glioblastoma cancer. This review showcases recent advancements in treatment strategies as well as possible future directions of these approaches to precision oncology and immunotherapy treatments for glioblastoma treatment [79].

As we seek to gain a better understanding of the extracellular mechanism of MS, equal emphasis should be given to preclinical research targeting the intracellular deposition of MS and its potential role in relieving neuronal dysfunction [80]. When conducting ongoing and future clinical investigations assessing immunotherapies for Parkinson’s disease, careful patient selection criteria, outcome criteria evaluation, and thorough pharmacodynamic assessments must all be implemented so as to accurately evaluate the efficacy of immunotherapies. 

Glioma, a leading primary intracranial malignancy, poses many difficulties due to limited treatment options and poor survival rates. Immunotherapy’s proven success with other cancers has opened the door for similar therapies that seek to activate patients’ immune systems to target and eliminate gliomas. Furthermore, this review highlights novel concepts and advanced technologies that hold promise in designing more effective immunotherapies, providing potential blueprints for more efficient glioma treatments [81]. 

Current treatments for advanced melanoma involve immunotherapy using anti-PD1 antibodies or targeted therapy with BRAF and MEK inhibitors, with immunomodulatory agents like LAG_3_, TIM_3_, OX_40_, CD_137_, IDO, and GITR being explored as potential therapeutic solutions. Research is being conducted into fully exploiting available treatments while simultaneously creating new drugs; however, an optimal first-line treatment remains uncertain [82].

Psychotropic medications have been employed to address psychiatric symptoms associated with NMDAR encephalitis; however, their effectiveness may be limited. There has been little investigation of their possible use as adjunct therapies to enhance glutamate and GABA neurotransmission. In this regard, it should be remembered that schizophrenia differs slightly from anti-NMDAR encephalitis, where there is an irreversible loss of surface NMDARs [83].

Future investigations should focus on understanding the significance of microglial subpopulation depletion across various disease stages. A comprehensive understanding is crucial before considering microglia depletion as a potential clinical intervention to resolve neuroinflammation and promote recovery, and genetic tools like CX3CR1CreER mice or targeted knockdown strategies can offer valuable insight. They may even aid in developing tailored therapeutic approaches [84].

## 8. Emerging Fields in Neuroimmunology

### 8.1. Neuroimmunology in Aging and Neurodegenerative Disorders

Chronic and acute stress can have profound impacts on health and functional wellbeing, with neuroimmunological dysregulation and inflammation serving as major contributing factors in numerous disease states [85]. Psychosocial strain and negative emotions have been associated with increased levels of pro-inflammatory biomarkers; immunosenescence—an aging process marked by declining immune function that leads to higher morbidity rates and susceptibility for fatal illnesses—particularly impacts neurodegenerative conditions like Parkinson’s disease and diabetes.

Recent studies on brain health and its effect on other body tissues remain incompletely understood; however, recent evidence points towards immune cells playing an integral part in brain aging. Studies of both aging mice and humans show an increase in type I interferon (IFN) response in the choroid plexus (CP), an area responsible for cognitive function and neurogenesis, and neutralizing age-related type I IFN responses partially restores cognitive function as well as hippocampal neurogenesis [86].

As individuals age, their brain’s immune cells, known as microglia, may become hyperreactive and display age-related pro-inflammatory biases. This phenomenon may be affected by both intrinsic factors (e.g., increased priming) and environmental ones, such as amplified danger signals, cytokines, or altered glymphatic function affecting microglia cells [87].

Aging and traumatic brain injuries (TBIs) may increase the priming of microglia, which leads to presymptomatic neurodegenerative diseases like Alzheimer’s. Risk increases with age and may also be affected by previous head trauma [88,89].

Aging has an adverse impact on the blood–brain barrier (BBB), rendering it more permeable and permitting increased immune activation, expression of stress-induced and inflammatory genes, and infiltration of CNS immune cells into the central nervous system (CNS). Such age-related changes in immunity could contribute to the ineffective clearance of toxic protein aggregates associated with neurodegenerative disorders [90].

### 8.2. Pediatric Neuroimmunology

Recent advancements in pediatric neuroimmunology have seen considerable advances. The detection of conformationally correct myelin oligodendrocyte glycoprotein (MOG) antibodies using cell-based assays has led to an increasing identification of children with various monophasic and relapsing phenotypes; however, debate remains as to the severity and outcome in this rapidly expanding spectrum of MOG-related demyelination [91].

Research efforts have primarily centered around studying Rituximab’s effects in pediatric neuromyelitis optica (NMO)/NMO spectrum disorders (NMOSD) patients and exploring its relationship to B-cell repopulation and relapses. Rituximab has proven effective at preventing relapses; however, the risk increases with B-cell repopulation—redosing before B-cell repopulation may help further decrease its likelihood [92].

Another area of investigation includes exploring how serum neurofilament light chain (sNfL) could serve as an accurate biomarker of disease activity and treatment response in pediatric patients with multiple sclerosis (MS). Studies suggest sNfL could serve as an accurate biomarker for monitoring disease activity and response among young MS patients, potentially helping predict severity levels as well as guide decision-making regarding treatment decisions in young MS cases [93].

Studies conducted among pediatric neuroimmunology populations demonstrated significant variance in 2-m and 4-m ETDRS chart scores, even after accounting for factors like optic neuritis (ON), vision correction, gender, and age, as revealed by the GEE model [94]. 

### 8.3. Neuroimmunoendocrinology: The Interactions between the Nervous, Immune, and Endocrine Systems

Bidirectional feedback communication among stress response, immune system, and endocrine system is integral for adaption to stressful stimuli, maintenance of homeostasis, and overall survival. Advancements have been made in understanding the molecular, cellular, and systemic physiological mechanisms underlying this communication—especially regarding immunity. Numerous neuroendocrine mediators like cortisol, estrogen, testosterone, DHEA catecholamines, corticotropin-releasing hormone, and adenosine have been identified as having immunomodulatory activities [95].

The nervous and immune systems share intimate connections, with close interactions regulating systemic homeostasis via the production and secretion of regulatory peptides (e.g., peptide hormones, cytokines, chemokines, and integrins). These molecules play an integral part in maintaining tissue homeostasis. Similarly, their production in both brain areas as well as central organs of immunity/endocrinology systems is comparable [96].

Maintaining homeostasis requires three regulating systems: nervous, endocrine, and immune. Interactions among these three have long been recognized, giving rise to neuroendocrinology. More recently, however, their combination is emerging as an exciting and rapidly expanding area of research [97].

These communication pathways encompass an array of interactions among cells, tissues, and organs with implications for mitochondrial functioning. Disruptions to interactions between mitochondria and neuroendocrine–immune systems contribute to Alzheimer’s and Parkinson’s diseases as pathologies [98]. 

Recent understandings of Parkinson’s disease have included immuno-inflammation and oxidative and nitrosative stress as key features, which are also seen in depression, somatization, and peripheral inflammation. We present evidence supporting their relevance in such conditions such as depression and somatization that are typically thought of as “comorbidities,” proposing instead that depression and somatization experienced throughout a lifetime, during the prodromal phase and concurrent with Parkinson’s, may actually play a significant role in its origins and progression rather than simply being “psychiatric” symptoms [99].

Interactions between neuroendocrine and immune systems have long been a focus of intense study and represent a highly promising area of investigation. Ample data are available that provide new insights into their bidirectional signal exchanges [100].

The hypothalamic–pituitary–adrenal (HPA) axis plays a key role in our body’s response to stress. Corticotropin-releasing factor (CRH), adrenocorticotropin (ACTH), and glucocorticoids play an integral part in stress response mechanisms, modulation of proinflammatory cytokines production, regulation of peripheral immune response regulation and neuroimmunoendocrine interactions regulating peripheral immunity response regulation; targeting this system could provide therapeutic approaches both human and experimental forms of Chagas disease [101]. 

### 8.4. The Role of Microbiota in Neuroimmunology

Recent interest in gut microbiota’s role in human health has renewed attention on its relationship to the brain–gut axis, yet its exploration dates back over three centuries [102]. We explore its historical development here with special reference to microbiota–neuroimmune communication, specifically highlighting that gut microbiota plays an integral part in pathogenicity for NMOSD (neuromyelitis optica spectrum disorder), suggesting microbiota interventions, such as diet changes, probiotics, antibiotics, or even fecal bacterial transplantation, could serve as treatment options for NMOSD patients [103].

Parkinson’s disease (PD) has long been linked with gut microbiota dysbiosis. Studies have demonstrated how imbalanced microbiota contributes to exacerbating symptoms. Germ-free (GF) animal models offer invaluable insight into this relationship between microbiota and various neurobiological and neurodevelopmental disorders—autism, obsessive compulsive disorder (OCD), depression, and anxiety, among others—and their microbiomes. GF rodents exhibit distinct behaviors when compared with conventional rodents that often exhibit anxiety- or depressive-like characteristics [103].

Th_17_ cells, an integral component of the immune response, can be stimulated by commensal microbiota present in the gut. Signaling between the nervous system and gastrointestinal tract appears to be impaired in conditions like irritable bowel syndrome (IBS), often described as a disorder of the gut–brain axis with multiple communication systems involved like microbiota–host crosstalk and neuroimmune interactions. Emerging evidence shows IBS patients possess different gut microbiomes than healthy individuals and, more specifically, dysbiotic colonic microbiota or mycobiota are found more frequently in those suffering from hypersensitivity [104]. 

Indeed, gut microbiota plays an indispensable role in regulating physiological and metabolic pathways. We will explore significant and current connections between gut microbiota and other systems in the body in subsequent sections. Any shifts in metabolic or physiological functions caused by changes to gut microbiota can create system-wide imbalances. Studies on interactions between host–microbiota interactions involving intestinal epithelium/immune system interactions have been extensively researched. Moreover, there has been increasing understanding regarding interactions between gut microbiota/neuroimmune system interactions, thus shedding light on their importance to overall health/wellbeing [105].

## 9. Conclusions

### 9.1. Summary of Key Findings

Multiple sclerosis (MS) research in neuroimmunology has brought forth significant advances, deepening our knowledge and improving treatment approaches for this condition:

(a) Immune Dysregulation: Multiple sclerosis is characterized by autoreactive T cells attacking the central nervous system, leading to inflammation and nerve fiber demyelination. Understanding their activation and regulation is crucial to discovering potential therapeutic targets for treatment;
(b) Cells Play an Important Role in Multiple Sclerosis Pathogenesis: B cells have emerged as key contributors to MS pathogenesis. By producing autoantibodies and modulating T-cell responses, these B cells play an integral part in tissue damage and neuroinflammation. Depletion therapies targeting B cells have shown promise as a possible treatment option for MS;
(c) Genetic Susceptibility: Genome-wide association studies have linked various genetic variations with MS susceptibility, enabling personalized therapy approaches and insights into genetic factors contributing to its cause;
(d) Environmental Triggers: Research has shed light on how environmental triggers like smoking, vitamin D deficiency, and viral infections interact to increase MS risk and severity. Researchers have also investigated the interaction between genetic predisposition and environmental influences in driving this process forward;
(e) Neuroprotective Strategies: Because MS leads to neurodegeneration, researchers are investigating neuroprotective measures that preserve nerve function and facilitate repair processes. Researching molecules involved with remyelination and axonal support could reveal therapeutic targets;
(f) Immunomodulatory Medications: Immune-system-targeted disease-modifying therapies have become a cornerstone of MS treatment, demonstrating significant slowing in disease progression and enhanced patient outcomes through early use.
(g) Studies have shed light on how gut microbiota influences immune responses and possibly contributes to MS. Understanding this relationship could lead to novel treatment approaches aimed at altering gut bacteria composition and functioning;
(h) Neuroimmunological studies have provided valuable insights into multiple sclerosis by shedding light on its gut–brain axis, genetic predispositions, environmental influences, B-cell involvement, immune dysregulation, and neuroprotective treatments as key elements. These discoveries hold great promise for developing more targeted and effective treatment strategies and ultimately improving the quality of life among those living with this complex neurological disorder.

### 9.2. Implications for Future Research

Future studies in neuroimmunology hold great promise for furthering our understanding of multiple sclerosis (MS) and developing more effective therapies. Key areas of investigation may include the following:

(a) Uncovering Underlying Mechanisms: Exploring the interrelations between MS’s immune and neurological systems can provide insight into specific physiological and molecular processes driving disease development while pinpointing key immune cell types, cytokines, and chemokines associated with inflammation and demyelination, which will allow targeted therapies;
(b) Neuroprotective Strategies: Although immune dysregulation is currently at the core of MS treatments, future research should prioritize neuroprotective approaches that preserve and restore damaged nerve cells. Deliberate identification of chemicals or pathways that promote neuronal survival and remyelination is key for slowing disability progression;
(c) Microbiome and Gut–Brain Axis: Understanding the role of gut microbiomes and gut–brain axis in MS pathophysiology can lead to innovative treatments that control immune responses and decrease disease activity, including interventions that regulate gut microbiomes;
(d) Precision Medicine: Advancements in molecular profiling, biomarker discovery, and tailored treatment strategies hold great potential to optimize medication selection treatment efficacy and minimize side effects in MS patients;
(e) Immunological Tolerance and Immunomodulation: Generating novel immunological tolerance mechanisms against myelin antigens could revolutionize MS therapy. Investigating innovative immunomodulatory strategies such as antigen-specific therapy or immune cell modulation could slow disease progression;
(f) Innovative Imaging Technologies: State-of-the-art imaging techniques like optical coherence tomography and advanced MRI methods offer more precise and early diagnostic indicators, monitor disease progression, and assess treatment efficacy in real time;
(g) Drug Repurposing: Investigating the therapeutic potential of existing neuroprotective or immunomodulatory medications can hasten the discovery of new MS treatments.

Future neuroimmunology research for MS should prioritize advanced imaging techniques, uncovering disease mechanisms, examining genetic and environmental influences, devising neuroprotective and precision medicine strategies, and exploring gut–brain axis/microbiome interactions while considering drug repurposing strategies—these efforts will allow for the creation of personalized, efficient therapies to enhance the quality of life for MS patients while lessening the burden of this debilitating condition.

### 9.3. Final Thoughts

An Exhaustive Analysis of Neuroimmunology: Lessons learned from multiple sclerosis and upcoming therapeutic advancements provides an essential resource on the complex interplay between immune and neurological systems, with multiple sclerosis (MS) as its focal point. It offers contemporary insight into its pathophysiology, clinical manifestations, and available treatment options, providing readers with a thorough overview.

This review offers invaluable insight into the neuroimmunological mechanisms underlying MS. It may address how immune cells, cytokines, and signaling molecules play a role in central nervous system inflammation responses and demyelination. Furthermore, genetic and environmental factors may play a part in contributing to its onset and its heterogeneous presentations in different patients’ cases.

One of the hallmarks of this comprehensive review is its detailed examination of current MS treatments. This likely involves discussing immunomodulatory drugs, symptom-relieving medication, and disease-modifying therapies. Furthermore, it may provide insight into emerging therapies or research methodologies that hold the promise of more precise medicines in the future.

As this review highlights, its primary message may be the importance of ongoing research and collaboration among neuroscientists, immunologists, and clinicians to fully comprehend neuroimmunology’s application in MS treatment. As our understanding of MS deepens, the potential exists for more tailored therapeutic advancements that could potentially enhance the quality of life for those living with MS.

Overall, this in-depth analysis serves as an indispensable resource for academics, medical professionals, and anyone wanting deeper insights into neuroimmunology and its application in treating multiple sclerosis today and in the future.

## Data Availability

All Data is available on PubMed.
